# Supplementary figures and images for: Isolation of the Buchnera aphidicola flagellum basal body complexes from the Buchnera membrane
Source: PLoS One. 2021 May 10;16(5):e0245710. doi: 10.1371/journal.pone.0245710 (PMC8109811; doi:10.1371/journal.pone.0245710)

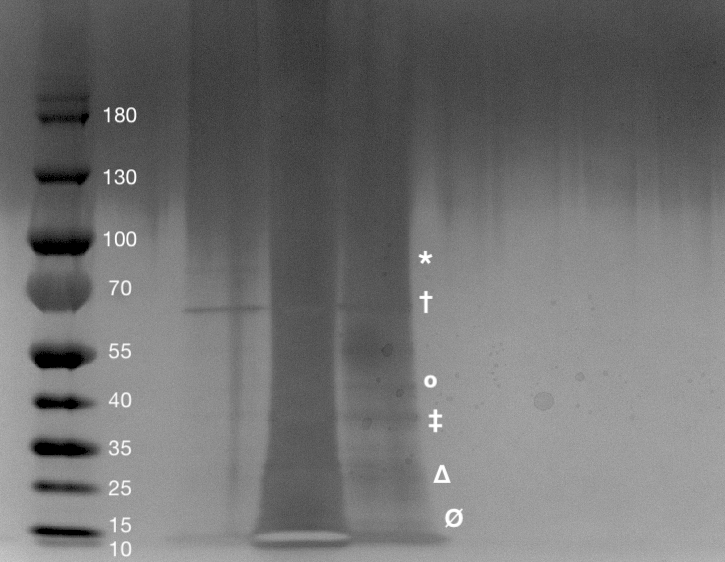

Supplement: S1 Fig — The first lane is taken directly from the enrichment preparation after overnight incubation with TET buffer. The second lane is after concentrating the enriched proteins to 1 mg/mL. The third lane is concentrated protein diluted to 0.5 mg/mL. Ladder values represent molecular weight in kDa. Symbols correspond to flagellar protein molecular weight: * corresponds to FlhA (78 kDa). † corresponds to FliF (63 kDa) and FlgK (63 kDa). ° corresponds to FlgE (45 kDa), FliP (43kDa), and FlgI (41 kDa). ‡ corresponds to FliG (38kDa) and FliM (37 kDa). Δ corresponds to FlgG (28 kDa), FlgF (28 kDa), FlgH (26 kDa), and FliH (26kDa). Ø corresponds to FlgB (16 kDa), FliN (15 kDa), FlgC (15 kDa), and FliE (11 kDa). (TIF) [file pone.0245710.s001.tif]

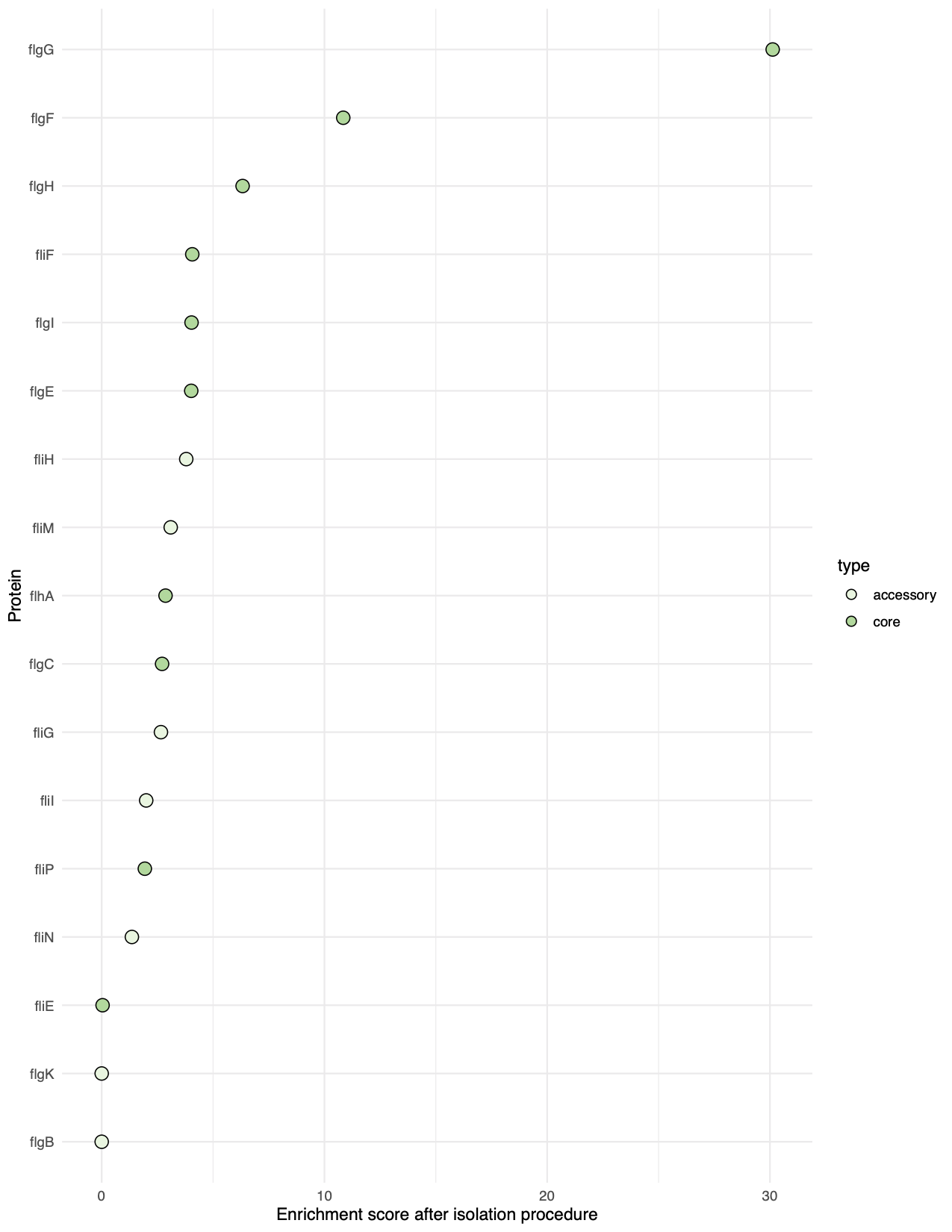

Supplement: S2 Fig — The enrichment score for each protein is indicated on the x axis. Enrichment scores are calculated by dividing unique spectral counts for each protein in the final step by each protein present in the cell lysate. Core flagellum proteins (defined by proteins required for type III secretion activity and flagellum structure) are filled in green, accessory proteins are filled in white. (TIFF) [file pone.0245710.s002.tiff]

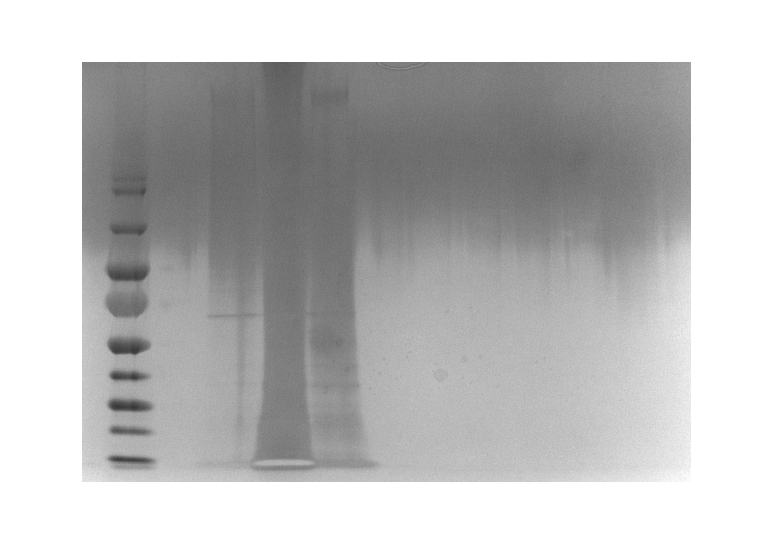

Supplement: S1 Original image — (TIF) [file pone.0245710.s005.tif]
