# Supplementary material for: Isolation of the Buchnera aphidicola flagellum basal body complexes from the Buchnera membrane
Source: PLoS One. 2021 May 10;16(5):e0245710. doi: 10.1371/journal.pone.0245710 (PMC8109811; doi:10.1371/journal.pone.0245710)
Supplement: S1 Protocol — (DOCX) [file pone.0245710.s004.docx]

**Extraction of flagellum basal body complexes from *Buchnera aphidicola,* an endosymbiont of aphids**

1. Grow aphids from birth to to fourth instar (10 days) and harvest from plants. For this protocol, we used 3-5g live aphids.
2. Load aphids into sterile, fine mesh tea infuser balls or other sterile straining device.
3. Submerge into 0.5% NaClO solution for 1 min.
4. Transfer and submerge into sterile MQ H_2_O at 4^o^C for 1 min.
5. Repeat step 4 (Transfer and submerge into sterile MQ H_2_O for 1 min).
6. Remove aphids from strainer, placing in a mortar kept at 4^o^C. Add 20mL buffer A + EDTA and homogenize aphids w/ pestle.
7. Pour homogenized aphids into sterile vacuum filter equipped with a 100uM nylon filter. Collect homogenate on ice.
8. Remove nylon filter and place aphid filter cake back into mortar. Repeat homogenization with an additional 20mL buffer A.
9. Repeat filtration with new 100mM nylon filter, combining filtrate.
10. Centrifuge 100uM filtrate for 10 min at 1500*g* at 4^o^C in a pre-cooled centrifuge.
11. Discard supernatant. Gently resuspend pellet in 40mL cold buffer A with sterile spatula or wide-bore pipette tip. Do not vortex.
12. Vacuum filter resuspension through a 20uM nylon filter. Collect filtrate on ice.
13. Immediately continue filtration through a 11uM nylon filter, collecting filtrate on ice.
14. Filter through a 5uM nylon filter, collecting filtrate on ice.
15. Centrifuge 5 uM filtrate for 30 min at 1500*g* at 4^o^C in a pre-cooled centrifuge.
16. Discard supernatant. Gently resuspend pellet in 100 mL sterile sucrose solution (0.5M sucrose, 100mM Tris-HCl [pH8.0]). Do not vortex.
17. Optional: Use light microscope to check integrity of *Buchnera* cells. Cells are spherical, averaging 3uM in size.
18. Begin to gently stir on ice.
19. Add 5mL lysozyme (2 mg/mL, freshly prepared in H2O at 4^o^C).
20. Add 10mL 100mM EDTA solution pH 7.5 and incubate on ice gently spinning for 40 min.
21. Add 10mL 10% Triton X-100 (in H2O) to lyse cells. Solution should turn from turbid to clear.
22. Once solution is transparent, add 100mM MgSO4 and 1 mg/ml RNase-free DNase. Incubate at room temperature, gently stirring, allowing DNases to degrade cellular DNA. Solution should become less viscous after 30 min, this step can be extended to an overnight incubation at 4^o^C if DNases require more time.
23. Raise pH of the solution to pH 10 with 1N NaOH.
24. Spin solution at 5000*g* in a pre-cooled 4C centrifuge three times, saving the supernatant and decanting into a new tube each time.
25. After three spins, transfer supernatant into a polyallomer centrifuge tube and spin at 30,000*g* for 1h at 4^o^C in a pre-cooled centrifuge.
26. Carefully decant or draw off supernatant and discarded. Cover pellet with TET (10mM Tris-HCl, 5mM EDTA, 0.1% Triton X-100, pH 8.0) and leave overnight at 4^o^C to soften protein pellet. *Buchnera* protein is ready for downstream analyses.

List of required reagents

**NaClO** 0.5% solution: 83.5 mL 6% NaCLO in 916.5 mL sterile H_2_O

**Buffer A**: 25mM KCl, 35mM Tris base, 10mM MgCl_2_, 250mM anhydrous EDTA in 950 mL sterile H_2_O. Add pellets of KOH until anhydrous EDTA is dissolved/solution becomes clear. Adjust pH to 7.5. Autoclave, then add 50 mL filter-sterilized 2.5M sucrose.

**Sucrose solution:** 0.5M sucrose, 100mM Tris-HCl. Raise pH to 8 and filter sterilize.

**Lysozyme solution:** Prepare 2 mg/mL egg white lysozyme in sterile H_2_O.

**EDTA solution:** 100mM anhydrous EDTA in sterile H_2_O. Add KOH until EDTA is dissolved/solution becomes clear. Adjust pH to 7.5.

**Triton X-100 solution:** 10% w/v Triton X-100 detergent in sterile H_2_O.

**MgSO4 solution**: 100mM MgSO_4_ in sterile H_2_O.

**RNase-free DNase:** 1 mg/mL in sterile H_2_O.

**NaOH, 1N:** Dissolve NaOH pellets in sterile H_2_O.

**TET Buffer:** 10mM Tris-Hcl, 5mM EDTA, 0.1% w/v Triton X-100. Add KOH or NaOH to dissolve anhydrous EDTA, then adjust pH to 8.0.
