# Supplementary material for: Isolation of the Buchnera aphidicola flagellum basal body complexes from the Buchnera membrane
Source: PLoS One. 2021 May 10;16(5):e0245710. doi: 10.1371/journal.pone.0245710 (PMC8109811; doi:10.1371/journal.pone.0245710)
Supplement: Attachment — Submitted filename: Schepers_response_final.docx [file pone.0245710.s006.docx]

**Comments to the Author**

3. Have the authors made all data underlying the findings in their manuscript fully available?

The [PLOS Data policy](http://www.plosone.org/static/policies.action%23sharing) requires authors to make all data underlying the findings described in their manuscript fully available without restriction, with rare exception (please refer to the Data Availability Statement in the manuscript PDF file). The data should be provided as part of the manuscript or its supporting information, or deposited to a public repository. For example, in addition to summary statistics, the data points behind means, medians and variance measures should be available. If there are restrictions on publicly sharing data—e.g. participant privacy or use of data from a third party—those must be specified.

Reviewer #1: Yes

Reviewer #2: No

The raw mass spec data is now uploaded to a public database, please see below.

5. Review Comments to the Author

Reviewer #1: The paper presents a procedure for isolating proteins for the flagellum basal body of Buchnera, which would facilitate further characterization of this enigmatic protein complex. I believe the paper is important and should be published after some minor edits.
My suggestions are as follows.
Title: Probably "basal body" should be replaced with "basal body proteins" or "basal body complexes". The original wording suggests that basal bodies can be isolated intact, which is not certain at this stage.

We agree and have changed the title as suggested.

p8, l9: "flagella" should be "flagellum basal body proteins". As the authors also describe, Buchnera lacks flagellin, meaning that it lacks flagella.

p8, l10-11: "flagellum proteins" and "flagellum structure" should also be corrected accordingly.

We agree and have changed the wording as suggested.

Introduction: In this section, I would like to see protein names and a corresponding figure similar to the current Figure 2.

We have modified the previous Fig. 2 to include this information. We show the genes both present and absent from the *Buchnera* genomes, and also include the information about enrichment.

p9, l19-20: Probably "flagellum proteins" should be "flagellum-related proteins".
p10, l14: Probably "basal bodies" should be replaced with "basal body proteins" or "basal body complexes".
p10, l22: Same as above.

We agree and have changed the wording as suggested.

p11, l3: Probably better to add "from Buchnera cells", which would clarify the meaning of "the start" in the following sentence. (Otherwise, it could be misunderstood as whole aphid homogenate.)

We agree and have changed the wording as suggested.

p11, l12: The fact that "basal bodies resemble top hats" is not a novel finding. I believe that the authors would like to say instead that they succeeded in obtaining apparently intact (?) basal bodies using the present procedure.

We agree and have changed the wording as suggested.

p11, l21: What "changes"?

We have clarified the wording.

p13, l9, "bleach solution": Better to show the compound name.

Fixed.

p13, l18: One more parenthesis is required after "Aldrich".

Fixed.

p13, l19, "remain alive": Better to show some evidence or references.

We have changed the wording to indicate the cells are still intact.

p17: A brief explanation about Tuf and GroL is required.

This was added.

p18: I believe this kind of figure should be shown in the introduction section.

As noted above, we have modified the figure and introduced it in the introduction.

Reviewer #2: In this manuscript, Schepers and co-authors report a protocol for isolating flagellar bodies from the membrane of Buchnera endosymbionts of aphids. Flagellar genes have been previously shown to be highly expressed in Buchnera and the flagellar basal bodies imaged on the surface of its cells, but the function of these structures for the aphid-Buchnera symbiosis remains unclear. Since the bacterial flagellum is homologous to the T3SS, these structures are often hypothesized to be involved in protein exchange between Buchnera cells and the host-derived (symbiosomal) membrane surrounding Buchnera’s double membrane. The manuscript only reports a protocol for isolating the flagellar basal bodies, so it is short and straightforward. My comments are relatively minor.

(1) Since the manuscript reports a new protocol, I would appreciate if the authors could upload a step-by-step protocol with as many details as possible to a server such as protocols.io (or to FigShare or as a supplementary file).

A step-by-step protocol has been provided as Supplementary File. We have uploaded a step-by-step protocol to [protocols.io](http://protocols.io), available at [dx.doi.org/10.17504/protocols.io.bs5wng7e](http://dx.doi.org/10.17504/protocols.io.bs5wng7e)

(2) If possible, please upload the raw mass spec data to a database or share them in any other way. I understand that sharing such data as supplementary material is not very common, but in this case I feel that having the data available would be beneficial to anyone trying to reproduce the protocol, e.g. to properly validate their results.

The raw mass spec data is now uploaded to the ProteomeXchange Consortium via the PRIDE repository as a Supplemental Dataset. Data are available via ProteomeXchange with identifier PXD024664.

(3) Figure 1. Were any aphid-derived or non-flagellar Buchnera proteins enriched after the isolation procedure?

No aphid proteins were enriched by this procedure. Other *Buchnera* proteins such as DNA-directed RNA polymerase subunits or membrane proteins such as components of the *sec-* secretion system were slightly enriched, but not to the extent of the flagellum-related proteins.

(4) Figure 3. Please provide more details about how many grids were visualized and how many top hat-like particles of flagellar complexes were approximately detected by TEM. Were any other macromolecular protein complexes enriched in the sample?

Other macromolecular protein complexes were not present (or easily identified) intact in TEM micrographs. TEM was limited due to time constraints and the institutional pandemic guidelines. 24 of 204 micrographs had at least one easily discernible partially-intact flagella complexes; we added this information.

5) Are there any other endosymbionts described with an incomplete flagellum (or T3SS) similar to the one known from Buchnera?

We have added a couple of sentences to the Discussion about the status of the flagellum complex in some endosymbionts other than *Buchnera*. Basically, it varies quite a bit but is often lost entirely.

Please correct 4C to 4ºC throughout the MS.

This has been fixed throughout.
